# Toll-like receptor 4 rs11536889 is associated with angiographic extent and severity of coronary artery disease in a Chinese population

**DOI:** 10.18632/oncotarget.14014

**Published:** 2016-12-18

**Authors:** Dandan Sun, Yupeng Wu, Honghu Wang, Hong Yan, Wen Liu, Jun Yang

**Affiliations:** ^1^ Department of Cardiovascular Ultrasound, The First Affiliated Hospital of China Medical University, Shenyang, China; ^2^ Department of Neurosurgery, The First Affiliated Hospital of China Medical University, Shenyang, China

**Keywords:** TLR4, polymorphism, coronary artery disease, extent and severity, Pathology Section

## Abstract

Toll-like receptor 4 (TLR4) is a key modulator in many inflammation-related diseases. Polymorphisms in the *TLR4* gene may alter TLR4 expression and affect the extent and severity of coronary artery disease (CAD). We analyzed 3 polymorphisms of *TLR4* in 607 Chinese subjects who underwent coronary arteriography. Blood samples were collected to identify the polymorphisms. We evaluated the relationships between the polymorphisms and the number of vessels involved in coronary stenosis, Gensini scores, and Duke prognostic scores. We found that rs11536889 was associated with an increased risk of 3-vessel disease. When subjects with 3-vessel disease were compared to subjects with nonsignificant CAD, rs11536889 variant genotypes were associated with an increased risk of 3-vessel disease (GC/CC *vs*. GG: OR=2.06, 95%CI=1.21-3.51). When subjects with 3-vessel disease were compared to subjects with 1-vessel disease, rs11536889 variant genotypes were associated with an increased risk of 3-vessel disease (GC *vs*. GG: OR=2.14, 95%CI=1.20-3.79; GC/CC *vs*. GG: OR=2.06, 95%CI=1.20-3.54). When subjects with 3-vessel disease were compared to subjects with non-3-vessel disease, rs11536889 variant genotypes were associated with an increased risk of 3-vessel disease (GC *vs*. GG: OR=1.76, 95%CI=1.12-2.75; GC/CC *vs*. GG: OR=1.83, 95%CI=1.19-2.82). The *TLR4* rs11536889 polymorphism was also related to Gensini score (*P*=0.02). The Gensini score was higher in subjects with the variant CC and GC/CC genotype than in subjects with the wild GG genotype (61.28 1.84 and 57.6434.82 *vs*. 51.2734.57). Our results demonstrate that *TLR4* rs11536889 polymorphism is a novel genetic factor in the development of CAD, influencing the extent and severity of CAD.

## INTRODUCTION

Coronary artery disease (CAD) is a leading cause of death worldwide. The extent and severity of CAD is correlated with mortality and longevity [1]. Substantial clinical data have illustrated that inflammation is an important etiological mechanism underlying CAD and contributes to the extent and severity of the disease [2].

Toll-like receptor (TLR) 4 is one of the most well-characterized inflammation-related molecules in the immune system; it is closely related to the extent and severity of CAD. Mizoguchi et al showed that TLR4 expression on peripheral venous blood monocytes was positively correlated with Gensini score in a CAD population [3]. Multiple studies have shown that TLR2 and TLR4 are upregulated in atherosclerotic plaques and circulating monocytes and that this enhanced expression is associated with more severe atherosclerotic diseases [4–6]. Moreover, the expression of inflammatory cytokines induced by TLR4 is significantly related to the degree of coronary stenosis within CAD patient groups [7].

As it is known, sequence variants especially the single nucleotide polymorphisms (SNPs) in the promoter region, may change the binding capacity of certain transcription factors, such as Sp1, or Nrf2 [8, 9]. Variants in the 3’-untranslated region (3’-UTR) may affect mRNA stability. All the above has hold great promise for altering *TLR4* transcription, and thus modulating disease susceptibility [10, 11]. Several studies have revealed that *TLR4* polymorphisms in the promoter region and 3’-UTR are associated with various inflammation-related diseases. Zhang et al reported a positive association between asthma severity and the GG genotype of *TLR4* rs10983755, which indicated that the variant A allele of rs10983755 has a protective effect on asthma severity [12]. A study by Minmin et al reported that individuals carrying the heterozygous genotype for the rs10116253 had a significantly decreased risk of hepatocellular carcinoma compared to those carrying the wild-type homozygous genotype [13]. Peng et al reported that the variant genotype of *TLR4* rs11536889 was associated with an increased risk of type 2 diabetes mellitus (DM) in female subjects, as well as high levels of 2-hour plasma glucose and high-density lipoprotein cholesterol [14].

No genetic study has investigated the relationship between *TLR4* polymorphisms in the promoter region and 3’-UTR and the extent and severity of CAD. In this study, we investigated the association between 3 tag-SNPs, which capture essential genetic information about the promoter region and 3’-UTR of the *TLR4* gene, and angiographically defined CAD severity and extent, from the number of vessels involved coronary stenosis, Gensini score to Duke prognostic score.

## RESULTS

### Characteristics of the study population

We included a total of 607 subjects who underwent coronary angiography in this study. The study population included 374 men (61.6%) and 233 women (38.4%). The mean age of the patients was 59.44 ± 9.80 years. Table 1 summarizes the characteristics of the subjects according to the number of vessels involved in coronary stenosis. In all, 106 subjects had nonsignificant CAD, 169 subjects had 1-vessel disease, 141 subjects had 2-vessel disease, and 111 subjects had 3-vessel disease. Compared to subjects in other groups, subjects with 3-vessel disease had a higher prevalence of dyslipidemia, hypertension, DM, and current use of cardiovascular medication (e.g,, statin, acetylsalicylic acid [ASA], and angiotension conversion enzyme inhibitor [ACEI]) (*P* < 0.05). Further, subjects with 3-vessel disease were older and had higher systolic blood pressure, total cholesterol levels, fasting plasma glucose levels, and 2-hour plasma glucose levels than subjects in other groups (*P* < 0.05). Sex, smoking status, and alcohol consumption were not different among different groups (*P* > 0.05).

**Table 1:** Patient characteristics by the number of vessels involved in coronary stenosis

Variable	Total	Nonsignificant CAD	1-vessel disease	2-vessel disease	3-vessel disease	
	n=607	n=186	n=169	n=141	n=111	*P*
Male(%)	374(61.6)	106(57.0)	105(62.1)	91(64.5)	72(64.9)	0.44
Female(%)	233(38.4)	80(43.0)	64(37.9)	50(35.5)	39(35.1)	0.44
Age(years)	59.44±9.80	59.72±9.12	56.78±9.65	60.47±10.52	61.68±9.41	**<0.01**
Dyslipidemia(%)	504(83.0)	147(78.7)	143(84.4)	113(79.9)	101(91.0)	**0.04**
CHO(mmol/L)	4.14±1.08	3.97±1.07	4.23±0.99	4.06±1.02	4.33±1.27	**0.03**
TRIGLY(mmol/L)	1.77±1.20	1.67±0.97	1.83±1.23	1.69±1.14	1.89±1.44	0.32
LDL-C(mmol/L)	2.58±0.94	2.47±0.94	2.63±0.84	2.50±0.94	2.74±1.04	0.08
HDL-C(mmol/L)	1.03±0.28	1.01±0.29	1.07±0.30	1.02±0.25	1.01±0.24	0.17
Hypertension(%)	420(69.1)	111(59.2)	118(69.7)	104(73.8)	87(78.4)	**<0.01**
SBP(mmHg)	159.94±34.46	151.58±33.71	160.37±32.06	165.12±37.91	165.28±32.85	**<0.01**
DBP(mmHg)	92.45±21.91	88.76±22.45	93.67±18.40	92.99±24.79	95.32±22.18	0.06
DM(%)	210(34.6)	54(29.0)	51(30.2)	48(34.0)	57(51.4)	**<0.01**
Fasting glucose(mmol/L)	6.38±1.99	6.03±1.50	6.30±1.87	6.42±1.97	7.04±2.67	**<0.01**
2h glucose(mmol/L)	9.57±4.38	8.72±3.41	9.78±4.91	9.13±4.19	10.93±4.68	**0.01**
Smoker(%)	231(37.9)	67(36.2)	61(36.1)	54(38.6)	49(44.1)	0.44
Drinker(%)	102(16.5)	37(20.0)	27(16.0)	18(12.8)	20(18.0)	0.29
Current medication use						
Statin(%)	422(69.6)	106(57.0)	120(71.0)	101(71.6)	95 (85.6)	**<0.01**
ASA(%)	355(58.5)	73(39.0)	102(60.6)	92(65.2)	88(78.9)	**<0.01**
ACEI(%)	319(52.5)	84(45.2)	90(53.3)	77(54.6)	72(64.8)	**<0.01**

Values are n(%) or mean±SD unless otherwise noted. CHO, cholesterol; TRIGLY, triglyceride; LDL-C, low-density lipoprotein cholesterol; HDL-C, high-density lipoprotein cholesterol; SBP, systolic blood pressure; DBP, diastolic blood pressure; DM, diabetes mellitus; ASA, acetylsalicylic acid; ACEI, angiotensin converting enzyme inhibitor.

Table S1 shows the distribution of *TLR4* tag-SNPs in the study population. The frequencies of each polymorphism within the population were in Hardy-Weinberg equilibrium (HWE) (*P* > 0.05). Table 2 displays the baseline clinical characteristics of the population according to genotype. No significant differences in traditional risk factors or laboratory parameters were observed among the genotype groups (*P* > 0.05).

**Table 2:** Patient characteristics by genotypes of TLR4 polymorphisms

Variable	rs10116253					rs10983755					rs11536889			
	TT	TC	CC	*P*		GG	GA	AA	*P*		GG	GC	CC	*P*
Male(%)	132(66.3)	182(59.1)	58(59.2)	0.23		185(64.5)	158(59.4)	30(56.6)	0.35		225(60.0)	128(65.0)	20(58.8)	0.48
Female(%)	67(33.7)	126(40.9)	40(40.8)	0.23		102(35.5)	108(40.6)	23(43.4)	0.35		150(40.0)	69(35.0)	14(41.2)	0.48
Age(years)	59.23±9.87	59.35±9.94	60.03±9.36	0.79		59.17±9.87	59.68±9.81	59.53±9.57	0.83		59.27±9.68	59.74±10.18	59.29±9.19	0.86
Dyslipidemia(%)	161(80.9)	262(85.1)	81(82.7)	0.46		233(81.2)	229(86.1)	42(79.2)	0.22		309(82.4)	169(85.8)	26(76.5)	0.33
CHO(mmol/L)	4.05±0.96	4.19±1.13	4.19±1.15	0.37		4.10±0.99	4.18±1.16	4.20±1.12	0.61		4.107±1.05	4.25±1.13	4.27±1.10	0.14
TRIGLY(mmol/L)	1.81±1.14	1.78±1.27	1.67±1.08	0.57		1.78±1.12	1.81±1.35	1.51±0.71	0.25		1.81±1.21	1.70±1.21	1.77±0.98	0.64
LDL-C(mmol/L)	2.50±0.88	2.62±0.94	2.62±1.03	0.32		2.54±0.88	2.62±0.96	2.58±1.06	0.61		2.53±0.92	2.66±0.95	2.65±0.98	0.27
HDL-C(mmol/L)	1.02±0.29	1.03±0.27	1.04±0.26	0.92		1.02±0.28	1.03±0.28	1.08±0.25	0.33		1.03±0.29	1.02±0.25	1.07±0.30	0.65
Hypertension(%)	141(70.9)	211(68.5)	67(68.4)	0.84		201(70.0)	182(68.4)	36(67.9)	0.90		259(69.1)	142(72.1)	18(52.9)	0.08
SBP(mmHg)	163.51±34.54	159.11±33.42	156.19±36.99	0.18		162.28±33.55	158.45±34.12	155.40±40.40	0.27		160.13±34.52	161.28±35.21	150.94±28.49	0.27
DBP(mmHg)	94.38±21.53	92.27±22.36	89.37±21.19	0.17		94.54±21.43	91.39±21.91	89.60±23.56	0.29		91.29±21.45	94.49±22.77	89.76±20.73	0.29
DM(%)	70(35.2)	98(31.8)	42(42.9)	0.13		106(36.9)	82(30.8)	22(41.5)	0.14		130(34.7)	68(34.5)	12(35.3)	0.99
Fasting glucose(mmol/L)	6.45±1.82	6.29±1.98	6.52±2.35	0.52		6.469±2.01	6.21±1.74	6.78±2.87	0.12		6.37±2.02	6.41±2.02	6.24±1.42	0.91
2-hour glucose(mmol/L)	9.67±4.47	9.30±4.21	10.28±4.73	0.36		9.69±4.61	9.40±4.16	9.72±4.27	0.84		9.89±4.62	9.36±4.13	7.69±2.61	0.07
Smoking(%)	76(38.2)	120(39.0)	34(34.7)	0.75		108(37.6)	103(38.7)	19(35.8)	0.91		133(35.4)	83(42.3)	14(41.2)	0.23
Drinking(%)	32(16.2)	54(17.5)	15(15.3)	0.80		42(14.6)	53(19.9)	6(11.3)	0.12		62(16.5)	37(18.9)	2(5.7)	0.08
Current medication use														
Statin(%)	145(72.9)	209(67.5)	68(68.7)	0.73		214 (74.6)	171(63.4)	38(9.6)	0.68		265(70.6)	138 (70.0)	19(55.9)	0.23
ASA(%)	111(55.8)	184(59.7)	60(61.2)	0.34		160(55.7)	163(61.3)	32(60.3)	0.42		210(56.0)	125(63.4)	20(58.8)	0.31
ACEI(%)	102(51.3)	166(53.9)	51(52.0)	0.49		147(51.2)	145(54.5)	27(50.9)	0.27		196(52.3)	107(54.3)	16(47.1)	0.41

Values are n(%) or mean±SD unless otherwise noted. TLR4, toll-like receptor 4; Other abbreviations as in Table 1.

### Effect of TLR4 polymorphisms on the number of vessels involved in coronary stenosis

The odds ratios (ORs) and 95% confidence intervals (CIs) for *TLR4* tag-SNPs and the number of vessels involved in coronary stenosis are shown in Table 3. We noted a significant association between the prevalence of 3-vessel disease and variant genotypes of *TLR4* rs11536889. When subjects with 3-vessel disease were compared to subjects with nonsignificant CAD, rs11536889 variant genotypes were associated with an increased risk of 3-vessel disease (GC/CC *vs*. GG: OR = 2.06, 95% CI = 1.21-3.51). When subjects with 3-vessel disease were compared to subjects with 1-vessel disease, rs11536889 variant genotypes were associated with an increased risk of 3-vessel disease (GC *vs*. GG: OR = 2.14, 95% CI = 1.20-3.79; GC/CC *vs*. GG: OR = 2.06, 95% CI = 1.20-3.54). When subjects with 3-vessel disease were compared to subjects with non-3-vessel disease, rs11536889 variant genotypes were associated with an increased risk of 3-vessel disease (GC *vs*. GG: OR = 1.76, 95% CI = 1.12-2.75; GC/CC *vs*. GG: OR = 1.83, 95% CI = 1.19-2.82). Variants in rs10116253 and rs10983755 were not related to the number vessels involved in coronary stenosis (*P* > 0.05) (Tables S2 and S3).

**Table 3:** Association between genotypes of TLR4 rs11536889 with the number of vessels involved in coronary stenosis

Group		GC vs. GG				CC vs. GG				GC/CC vs. GG				CC vs. GC/GG		
		OR(95%CI) ^a^	*P* ^a^	*P* ^b^		OR(95%CI) ^a^	*P* ^a^	*P* ^b^		OR(95%CI) ^a^	*P* ^a^	*P* ^b^		OR(95%CI) ^a^	*P* ^a^	*P* ^b^
1-vessel disease vs. nonsignificant CAD		0.75(0.46, 1.20)	0.23	0.68		2.61(0.88, 7.75)	0.08	0.25		0.87(0.56, 1.36)	0.54	0.99		2.53(0.86, 7.46)	0.09	0.28
2-vessel disease vs. nonsignificant CAD		0.81(0.50, 1.32)	0.40	0.99		2.21(0.65, 7.51)	0.21	0.62		0.90(0.56, 1.43)	0.64	0.99		2.05(0.62, 6.77)	0.24	0.71
3-vessel disease vs. nonsignificant CAD		1.93(1.11, 3.37)	0.02	0.06		2.77(0.89, 8.65)	0.08	0.24		**2.06(1.21, 3.51)**	**0.01**	**0.02**		2.23(0.74, 6.70)	0.15	0.46
2-vessel disease vs. 1-vessel disease		1.12(0.67, 1.89)	0.66	0.99		0.82(0.30, 2.24)	0.69	0.99		1.06(0.65, 1.73)	0.81	0.99		0.78(0.29, 2.09)	0.62	0.99
3-vessel disease vs. 1-vessel disease		**2.14(1.20, 3.79)**	**0.01**	**0.03**		1.65(0.60, 4.50)	0.33	0.99		**2.06(1.20, 3.54)**	**0.01**	**0.03**		1.20(0.45, 3.18)	0.71	0.99
3-vessel disease vs. 2-vessel disease		1.44(0.85, 2.43)	0.17	0.52		3.90(1.15, 13.24)	0.03	0.09		1.59(0.96, 2.63)	0.07	0.21		3.13(0.96, 10.25)	0.06	0.18
3-vessel disease vs. non 3-vessel disease		**1.76(1.12, 2.75)**	**0.01**	**0.04**		2.57(1.07, 6.17)	0.03	0.10		**1.83(1.19, 2.82)**	**0.01**	**0.02**		1.96(0.84, 4.55)	0.12	0.35

a, these tests were adjusted by age, sex, hypertension, diabetes mellitus, dyslipidemia, current medication use, smoking status and drinking status; b, P value was corrected by Bonferroni method; TLR4, toll-like receptor 4.

### Effect of TLR4 polymorphisms on Gensini score

**Figure 1:**
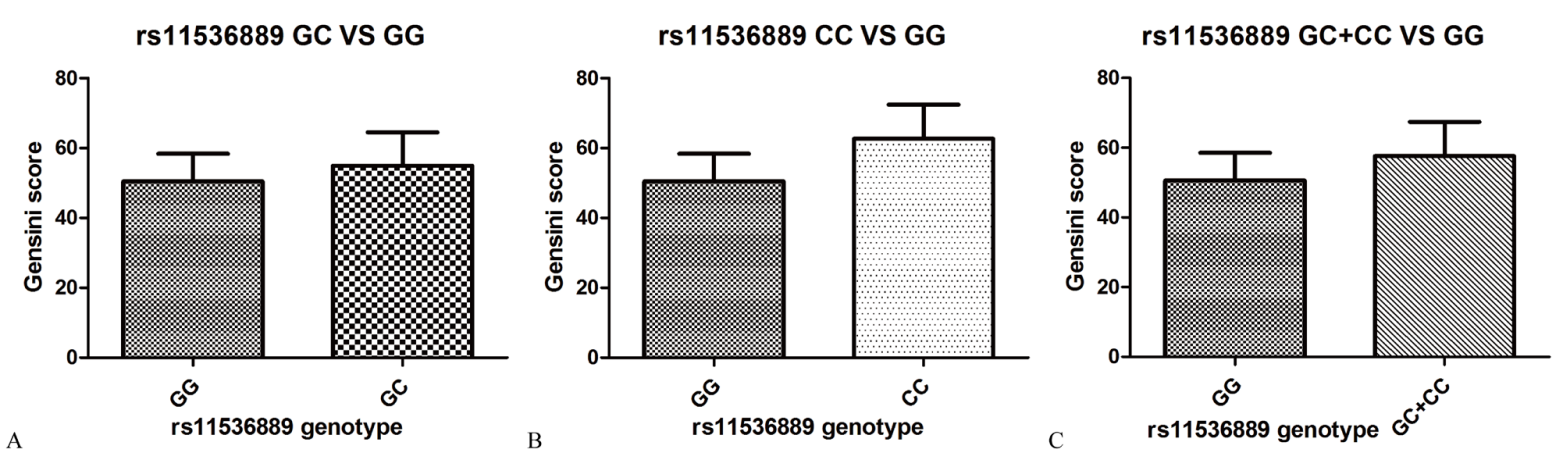
The extent and severity of CAD by TLR4 rs11536889 genotype **A**. The Gensini score between GC and GG genotype. **B**. The Gensini score between CC and GG genotype. **C**. The Gensini score between GC/CC and GG genotype. *P* value of log Gensini score and rs11536889 is 0.02. The model included age, sex, dyslipidemia, hypertension, DM, smoking status, alcohol consumption, current medication use, rs10116253, rs10983755 and rs11536889. The risk allele is C. CAD, coronary artery disease; TLR4, toll-like receptor 4; DM, diabetes mellitus.

Table 4 summarizes the results of *TLR4* tag-SNPs association analyses with Gensini scores of CAD in the studied population. The Gensini score of subjects in the variant CC genotype group and the GC/CC genotype group were greater than the score of subjects in the wild GG genotype group (61.28 ± 31.84 and 57.64 ± 34.82 *vs*. 51.27 ± 34.57; *P* = 0.01 and 0.03, respectively) (Figure 1). The association between rs11536889 and Gensini score remained significant after adjusting for other confounding factors (*P* = 0.02) (Table 5). *TLR4* rs10116253 and rs10983755 were not significantly associated with Gensini score (*P* > 0.05) (Tables 4 and 5).

**Table 4:** Association of genotypes of TLR4 rs11536889 with Gensini Score and Duke prognostic score

Polymorphism	Genotype	Gensini score			Duke prognostic score	
		Mean±SD	*P*		Mean±SD	*P*
rs10116253	TT	56.30±34.24	ref		36.49±25.15	ref
	TC	51.27±34.24	0.11		32.93±26.41	0.13
	CC	55.49±37.05	0.85		34.31±26.11	0.49
	TC/CC	52.35±34.98	0.18		33.26±26.31	0.15
rs10983755	GG	55.49±34.65	ref		36.57±26.31	ref
	GA	51.77±35.15	0.21		32.70±25.53	0.10
	AA	53.42±33.08	0.69		35.47±25.18	0.80
	GA/AA	50.02±34.73	0.22		34.32±25.47	0.15
rs11586889	GG	51.27±31.84	ref		32.50±25.11	ref
	GC	56.98±34.57	0.10		36.14±27.03	0.11
	CC	61.28±35.31	**0.01**		44.09±26.35	**0.03**
	GC/CC	57.64±34.82	**0.03**		37.31±27.02	0.07

TLR4, toll-like receptor 4.

**Table 5:** Multivariate predictors of the extent and severity of CAD

Characteristic	Log Gensini Score				Log Duke Score		
	B	SE	*P*		B	SE	*P*
Age (years)	0.01	0.01	0.25		0.01	0.01	0.07
Sex	-0.10	0.03	< 0.01		-0.03	0.02	0.25
Dyslipidemia	-0.01	0.04	0.74		-0.03	0.03	0.26
Hypertension	-0.03	0.03	0.27		-0.04	0.02	0.06
DM	-0.13	0.03	< 0.01		-0.07	0.02	< 0.01
Smoker	-0.02	0.04	0.50		-0.03	0.02	0.19
Drinker	0.07	0.04	0.09		0.05	0.03	0.09
Statin use	-0.03	0.04	0.33		-0.03	0.02	0.12
ASA use	-0.03	0.04	0.52		-0.03	0.03	0.29
ACEI use	-0.02	0.03	0.81		-0.03	0.03	0.29
TLR4 rs10116253	0.01	0.03	0.98		-0.02	0.02	0.39
TLR4 rs10983755	0.01	0.03	0.96		0.02	0.02	0.33
TLR4 rs11536889	-0.06	0.03	**0.02**		-0.03	0.02	0.10

TLR4, toll-like receptor 4; CAD, coronary artery disease; SNP, single nucleic polymorphism.

### Effect of TLR4 polymorphisms on Duke prognostic score

*TLR4* rs11536889 was associated with higher Duke prognostic score (Table 4). The highest Duke prognostic score was observed in subjects with the variant GC/CC genotype of *TLR4* rs11536889 (44.09 ± 26.35); the lowest score was observed in subjects with the wild GG genotype of *TLR4* rs11536889 (32.50±25.11) (*P* = 0.03). The association between rs11536889 and Duke prognostic score became insignificant after adjusting for other confounding factors (*P* > 0.05) (Table 5). There were no relationships between *TLR4* rs10116253 or rs10983755 and the Duke prognostic score (*P* > 0.05) (Tables 4 and 5).

## DISCUSSION

This is the first study to report that a 3’-UTR polymorphism (rs11536889) of *TLR4* is related to the extent and severity of CAD. According to our data, subjects with variant genotypes of *TLR4* rs11536889 had a higher risk of developing 3-vessel disease than the subjects with the wild-type genotype GG. We also noted a robust, direct relationship between variant genotypes of *TLR4* rs11536889 and Gensini score.

TLR4 is involved in the inflammatory process, which plays a vital role in the progression of atherosclerotic cardiovascular disease. First, TLR4 can induce the proliferation of vascular smooth muscle cells and upregulate the expression of the elastin-degrading enzyme and cathepsin S that allow smooth muscle cells to migrate to the site of atherosclerosis [15, 16]. Second, Vink et al found that stimulation of TLR4 on adventitial fibroblasts augmented neointima formation, an effect that was reduced in TLR4-defective mice by using a mouse femoral cuff model [17]. Third, several descriptive studies have showed that TLR4 is expressed by macrophages in murine and human lipid-rich atherosclerotic plaques [4, 18]. The activated TLR4 on macrophage can initiate a signal cascade that induces expression of inflammatory cytokines and proteases; this process may play a role in the formation and modeling of advanced atherosclerotic lesions [19].

In the present study, we demonstrated that *TLR4* rs11536889 is associated with 3-vessel disease: the variant genotypes are risk factors for more extensive and severe CAD. Many previous studies have suggested that genetic variation of rs11536889 may influence human inflammation-related diseases [20–23]. The GC or CC genotypes of *TLR4* rs11536889 have been associated with severe gastric atrophy in Japanese subjects who are *Helicobacter pylori* seropositive [20]. Castano-Rodriguez et al found that the *TLR4* rs11536889 C allele was a risk factor for gastric cancer in Chinese subjects [21]. Wang et al found a trend toward a higher frequency of GC/CC genotypes in patients with sepsis, and the C allele was significantly associated with susceptibility to sepsis [22]. Another large study including 1383 prostate cancer patients and 780 age-matched controls in Sweden revealed that the frequencies of the variant genotypes of *TLR4* rs11536889 were significantly higher in patients (24.1%) than in controls (19.7%) [23]. Until now, the correlation between *TLR4* rs11536889 and CAD with multiple vessel involvement has not been evaluated. However, one study reported that the presence of the variant C allele of *TLR4* rs11536889 loses a motif binding for hsa-miR-1236 and hsa-miR-642a, which can specifically bind to target mRNA. This variant allele results in translational inhibition, thus increasing the expression of TLR4 [24]. Furthermore, peripheral blood mononuclear cells from the CC and GC subjects secreted higher levels of IL-8 in response to TLR4 ligand than the cells from the GG subjects [25]. Our results are consistent with the theory that *TLR4* rs11536889 variant genotypes act as a risk factor for atherosclerosis development via increased TLR4 production. Therefore, our results raise the possibility that subjects with variant risk genotypes of *TLR4* rs11536889 acquire a greater atheromatous burden in the development phase of CAD.

The Gensini score describes not only the number of stenosed vessels but also the percentage of narrowing of the major vessels and the anatomical location of the stenosis [26]. This score is often used for quantifying the extent and severity of CAD. Among the subjects in our population, we observed an association between *TLR4* rs11536889 polymorphism and Gensini scores: the variant GC/CC genotypes of *TLR4* rs11536889 were associated with a higher Gensini score. Several experimental studies have shown that altered production of TLR4 appears to be critical for the evolution and progression of the atherosclerotic process. Hollestelle et al evaluated TLR4 in a mouse model: after ligation of the femoral artery, they observed a paradoxical increase in wall thickness accompanied by a hyperplastic response of the arterial wall. This phenomenon was not detected in TLR4-deficient mice [27]. Recently, Yin et al showed that TLR4 mediated intracellular lipid accumulation and ultimately led to foam cell formation by upregulating the expression of Acyl-coenzyme A: cholesterol acyltransferase 1, an intracellular enzyme that converts free cholesterol into cholesteryl esters for storage in lipid droplets [28]. The above evidence, together with our data, suggest the possibility that, in the process of atherosclerotic remodeling of adult human vessels, alterations in TLR4 production resulting from rs11536889 in the 3’-UTR of the *TLR4* gene could have substantial impacts on the extent and severity of CAD.

Our study has some potential limitations. First, the results of our study may be affected by selection bias. However, the genotype distribution of subjects was compatible with HWE. Second, the relatively small sample size may under power our results. Third, additional molecular biology studies evaluating the expression of TLR4 are necessary to identify the biological effect of the rs11536889 polymorphism of *TLR4*. Last, our study was performed in a Chinese Han population. Our findings should be confirmed in other geographic regions and ethnic groups.

In conclusion, in this cohort of 607 Chinese subjects with CAD, we demonstrated that variant genotypes of *TLR4* rs11536889 were associated with an increased risk of developing 3-vessel disease and a higher Gensini score. *TLR4* rs11536889 is a novel genetic factor in the development of CAD and significantly influences the extent and severity of CAD.

## MATERIALS AND METHODS

### Study population

We enrolled 607 consecutive subjects who underwent coronary angiography at the First Affiliated Hospital of China Medical University between December 2012 and January 2016. This study was approved by the Ethics Committee of China Medical University, and carried out in accordance with the Declaration of Helsinki. Written informed consent was obtained from each subject. Subjects were excluded from participation if they had a history of percutaneous coronary intervention or coronary artery bypass surgery, a history of malignant disease, an autoimmune disease, or cardiomyopathy [29]. Clinical data included sex, age, hypertension, dyslipidemia, DM, current medication use (e.g., statins, ASA, and ACEIs), smoking status, and alcohol consumption. A “smoker” was defined as a subject who smoked at least one cigarette per day for more than one year; a “drinker” was defined as a subject who consumed at least one alcoholic drink per day for a minimum of 6 months.

### Angiography

All of the coronary angiograms were reviewed by 2 experienced cardiologists who were both blinded to the genotype results. The extent and severity of CAD was estimated according to the results of the coronary angiography, including the number of vessels involved in the coronary stenosis and quantitative angiographic scores (i.e., Gensini score and Duke prognostic score). Detailed criteria for the evaluation methods are list in Table S4 [30–32].

### Genotyping

A 2-step approach, which has been described previously, was performed to identify the genotype of the 3 tag-SNPs [33]. Briefly, genomic DNA was obtained from peripheral blood using the standard phenol-chloroform method. Next, the polymorphisms were detected using the polymerase chain restriction-restriction fragment length polymorphism (PCR-RFLP) procedure. Table S5 lists the details of the PCR-RFLP conditions for the 3 tag-SNPs.

### Statistical analysis

Continuous variables were presented as mean ± standard deviation, and categorical variables were presented as numbers and percentages. Differences in baseline characteristics among groups were evaluated using analysis of variance (ANOVA), student's t-test, or the chi-square test. HWE in the study population was evaluated by the chi-square test.

The associations between the number of vessels involved in coronary stenosis (i.e., the proportions of patients with nonsignificant CAD, 1-vessel disease, 2-vessel disease, non-3-vessel disease, and 3-vessel disease) and *TLR4* SNPs (i.e., variant heterozygote genotype, variant homozygote genotype, dominant model, and recessive model) were compared using multiple logistic regression tests. The models were adjusted according to sex, age, hypertension, dyslipidemia, DM, current medication use, smoking status, and alcohol consumption [34]. *P* values were corrected for multiple comparisons using the Bonferroni method.

One-way ANOVA was used to determine the differences of quantitative angiographic scores among genotype groups. Logistic and linear regression models were constructed to test the additive effects of the SNPs on the extent and severity of CAD (i.e., log Gensini Score and log Duke score); each SNP was coded as 2, 1, or 0 according to the number of risk alleles. The model included age, sex, dyslipidemia, hypertension, DM, smoking status, alcohol consumption, current medication use, rs10116253. rs10983755 and rs11536889 [35]. A 2-tailed *P* value less than 0.05 was considered significant. All statistical analyses were performed using SPSS 16.0 Software (Chicago, IL, USA).

## SUPPLEMENTARY MATERIALS FIGURES AND TABLES


