# Supplementary material for: Toll-like receptor 4 rs11536889 is associated with angiographic extent and severity of coronary artery disease in a Chinese population
Source: Oncotarget. 2016 Dec 18;8(2):2025–33. doi: 10.18632/oncotarget.14014 (PMC5356775; doi:10.18632/oncotarget.14014)
Supplement: Supplementary file 1 [file oncotarget-08-2025-s001.pdf]

Toll-like receptor 4 rs11536889 is associated with angiographic extent and severity of coronary artery disease in a Chinese population

Supplementary Material

Table S1: The genotype frequencies and HWE in this study.

SNP	alleles	Major	Heterozygous	Minor	MAF	*P* of HWE	missing
rs10116253	T>C	207(33.0)	316(50.3)	103(16.4)	0.42	0.63	2
rs10983755	G>A	301(47.9)	272(43.3)	54(8.6)	0.30	0.80	1
rs11536889	G>C	388(61.8)	204(32.5)	35(5.6)	0.22	0.50	1

Values are n(%) unless otherwise noted. SNP, single nucleic polymorphism; MAF, minor allele frequency; HWE, Hardy-Weinberg equilibrium.

Table S2: Association between genotypes of TLR4 rs10116253 with the number of vessels involved in coronary stenosis.

Group	TC vs. TT			CC vs. TT			TC/CC vs. TT			CC vs. TC/TT		
	OR(95%CI) [a]	*P* [a]	*P* [b]	OR(95%CI) [a]	*P* [a]	*P* [b]	OR(95%CI) [a]	*P* [a]	*P* [b]	OR(95%CI) [a]	*P* [a]	*P* [b]
1-vessel disease vs. nonsignificant CAD	0.91(0.56, 1.48)	0.70	0.99	0.95(0.48, 1.89)	0.89	0.99	0.92(0.58, 1.46)	0.73	0.99	0.95(0.52, 1.72)	0.86	0.99
2-vessel disease vs. nonsignificant CAD	0.92(0.56, 1.52)	0.76	0.99	0.86(0.43, 1.73)	0.68	0.99	0.91(0.57, 1.47)	0.71	0.99	0.95(0.52, 1.77)	0.88	0.99
3-vessel disease vs. nonsignificant CAD	0.82(0.47, 1.44)	0.49	0.99	1.33(0.64, 2.76)	0.45	0.99	0.94(0.55, 1.59)	0.81	0.99	1.50(0.79, 2.85)	0.21	0.64
2-vessel disease vs. 1-vessel disease	0.89(0.53, 1.50)	0.66	0.99	0.85(0.41, 1.76)	0.67	0.99	0.88(0.54, 1.45)	0.63	0.99	0.89(0.46, 1.72)	0.74	0.99
3-vessel disease vs. 1-vessel disease	0.79(0.43, 1.46)	0.45	0.99	1.27(0.59, 2.75)	0.54	0.99	0.91(0.52, 1.59)	0.73	0.99	1.39(0.70, 2.77)	0.35	0.99
3-vessel disease vs. 2-vessel disease	0.89(0.49, 1.60)	0.69	0.99	1.30(0.62, 2.76)	0.49	0.99	0.97(0.56, 1.68)	0.92	0.99	1.34(0.68, 2.63)	0.39	0.99
3-vessel disease vs. non 3-vessel disease	0.89(0.55, 1.45)	0.65	0.99	1.32(0.72, 2.41)	0.37	0.99	0.98(0.63, 1.55)	0.94	0.99	1.36(0.80, 2.34)	0.26	0.78

[a], these tests were adjusted by age, sex, hypertension, diabetes mellitus, dyslipidemia, current medication use, smoking status and drinking status; [b], *P* value was corrected by Bonferroni method; TLR4, toll-like receptor 4.

Table S3: Association between genotypes of TLR4 rs10983755 with the number of vessels involved in coronary stenosis.

Group	GA vs. GG			AA vs. GG			GA/AA vs. GG			AA vs. GA/GG		
	OR(95%CI) [a]	*P* [a]	*P* [b]	OR(95%CI) [a]	*P* [a]	*P* [b]	OR(95%CI) [a]	*P* [a]	*P* [b]	OR(95%CI) [a]	*P* [a]	*P* [b]
1-vessel disease vs. nonsignificant CAD	0.82(0.53, 1.30)	0.40	0.99	1.44(0.63, 3.31)	0.39	0.99	0.89(0.58, 1.37)	0.59	0.99	1.47(0.66, 3.26)	0.35	0.99
2-vessel disease vs. nonsignificant CAD	0.71(0.44, 1.14)	0.16	0.47	1.56(0.69, 3.56)	0.29	0.86	0.81(0.52, 1.26)	0.34	0.99	1.86(0.84, 4.10)	0.13	0.38
3-vessel disease vs. nonsignificant CAD	0.87(0.52, 1.46)	0.60	0.99	1.44(0.52, 3.98)	0.48	0.99	0.91(0.56, 1.50)	0.72	0.99	1.44(0.55, 3.76)	0.45	0.99
2-vessel disease vs. 1-vessel disease	0.83(0.50, 1.36)	0.45	0.99	1.10(0.50, 2.39)	0.82	0.99	0.87(0.55, 1.39)	0.57	0.99	1.18(0.55, 2.51)	0.68	0.99
3-vessel disease vs. 1-vessel disease	1.08(0.62, 1.87)	0.79	0.99	0.70(0.26, 1.87)	0.48	0.99	0.99(0.58, 1.68)	0.97	0.99	0.66(0.26, 1.68)	0.38	0.99
3-vessel disease vs. 2-vessel disease	1.26(0.73, 2.18)	0.40	0.99	0.71(0.28, 1.77)	0.46	0.99	1.12(0.67, 1.88)	0.67	0.99	0.62(0.26, 1.50)	0.29	0.86
3-vessel disease vs. non 3-vessel disease	1.04(0.66, 1.62)	0.87	0.99	0.93(0.42, 2.08)	0.86	0.99	1.01(0.66, 1.55)	0.96	0.99	0.89(0.41, 1.92)	0.76	0.99

[a], these tests were adjusted by age, sex, hypertension, diabetes mellitus, dyslipidemia, current medication use, smoking status and drinking status; [b], *P* value was corrected by Bonferroni method; TLR4, toll-like receptor 4.

Table S4: The detailed criteria for each evaluation method.

1. Number of coronary stenosis

Nonsignificant CAD: < 50% stenosis in ≥ 1 epicardial vessel

1-vessel disease: ≥ 50% stenosis in one major vessel (LAD, LCX, RCA)

2-vessel disease: ≥ 50% stenosis in two major vessel or in the LMCA

3-vessel disease: ≥ 50% stenosis in three major vessel or in one major epicardial vessel and LMCA

2. Gensini score:

Reduction of 25%, 50%,75%, 90%, 99%, and complete occlusion values were given score of 1, 2, 4, 8, 16, and 32, respectively.

To each principal vascular segment was assigned: LMCA, × 5; proximal segment of LAD, × 2.5; proximal segment of LCX, × 2.5; mid-segment of LAD, × 1.5; RCA, distal segment of LAD, distal segment of LCX, PDA, D1, OM1, OM2, × 1; and others, × 0.5.

3. Duke prognostic score:

0: No CAD (≥50%)

19: 1-vessel disease (50%-74%)

23: > 1-vessel disease (50%-74%) or 1-vessel disease (75%)

32: 1-vessel disease (≥95%)

37: 2-vessel disease (≥50%)

42: 2-vessel disease (both≥95%)

48: 1-vessel disease (≥50%), including ≥95% proximal LAD or 2-vessel disease (≥50%), including ≥95% LAD

56: 2-vessel disease (≥50%), including ≥95% proximal LAD or 3-vessel disease (≥50%)

63: 3-vessel disease (≥50%), ≥95% in at least one

67: 3-vessel disease (≥50%), including 75% proximal LAD

74: 3-vessel disease (≥50%), including ≥95% proximal LAD

82: LMCA (75%)

100: LMCA (≥95%)

CAD, coronary artery disease; LAD, left anterior descending artery; LCX, left circumflex artery; RCA, right coronary artery; LMCA, left main coronary artery; PDA, posterior descending artery; D1, the first diagonal branch; OM1, the first obtuse marginal branch; OM2, the second obtuse marginal branch.

Table S5: Primer sequence and reaction condition.

Varible	TLR4 tag-SNPs		
	rs10116253	rs10983755	rs11536889
Forward and Reverse primers	F: GGGTGTAAAAGCCAGGTAGAGGAGGT<br>R: TGGAAAGTAGCAAGTGCAATGTAAGT	F: TGCCAGAACATCAAGAACACAGAAAAG<br>R: TGGAAAGTAGCAAGTGCAATGTAAGT	F: GCAGGAAGGAAGTGGGATGAC<br>R: TGTTTCTGAGGAGGCTGGATG
Annealing temperature (℃)	63	60	63
Product size (bp)	601	463	391
Restriction endonuclease	BsmAI	TaiI	TaaI
Digestion temperature (℃)	37	65	65
Fragment size (bp)	601–470–131	463–342–121	391–292–99

TLR4, toll-like receptor 4; SNP, single nucleic polymorphism.
